# Ultrasound Imaging of Ankle Retinacula: A Comprehensive Review

**DOI:** 10.3390/tomography10080095

**Published:** 2024-08-14

**Authors:** Carmelo Pirri, Nina Pirri, Veronica Macchi, Andrea Porzionato, Raffaele De Caro, Carla Stecco

**Affiliations:** 1 Department of Neurosciences, Institute of Human Anatomy, University of Padova, 35121 Padova, Italy; veronica.macchi@unipd.it (V.M.); andrea.porzionato@unipd.it (A.P.); rdecaro@unipd.it (R.D.C.); carla.stecco@unipd.it (C.S.); 2 Department of Medicine—DIMED, School of Radiology, Radiology Institute, University of Padova, 35122 Padova, Italy; nina_92_@hotmail.it

**Keywords:** ankle, foot, retinacula, deep fascia, ultrasound, imaging, ultrasonography, anatomy, ultrasound examination, peroneal retinaculum, superior extensor retinaculum, flexor retinaculum

## Abstract

The retinacula of the ankle are specialized anatomical structures characterized by localized thickenings of the crural fascia that envelop the deep components of the lower leg, ankle and foot. The ankle retinacula include the extensor retinacula, the peroneal retinacula and flexor retinaculum. Despite their potential to explain persistent and unexplained pain following an injury, these structures are often overlooked or incorrectly diagnosed. Hence, this comprehensive review was performed aiming to investigate the use and the methodology of US imaging to assess ankle retinacula. The search was performed on PubMed and Web of Science databases from inception to May 2024. The MeSH keywords used were as follows: “Ankle Retinacula”, “Foot Retinacula”, “Superior extensor retinaculum”, “Inferior extensor retinaculum”, “peroneal retinaculum”, “superior peroneal retinaculum”, “inferior peroneal retinaculum”, “flexor retinaculum”, “Ultrasound Imaging”, “Ultrasound”, “Ultrasonography” and “Ultrasound examination”. In total, 257 records underwent screening, resulting in 22 studies meeting the criteria for inclusion after the process of revision. Data heterogeneity prevents synthesis and consistent conclusions. The results showed that advanced US imaging holds promise as a crucial tool to perform an US examination of ankle retinacula, offering static and dynamic insights into ankle retinacula pathology. Understanding normal anatomy and US imaging is essential for accurately identifying injuries. Future research should focus on clinical trials to validate parameters and ensure their reliability in clinical practice.

## 1. Introduction

Musculoskeletal ultrasound (MSK US) imaging plays a pivotal role in the diagnosis and management of musculoskeletal disorders [1]. Additionally, MSK US is a non-invasive, radiation-free and cost-effective tool, making it accessible for routine use in clinical practice [1]. Its utility extends to guiding therapeutic interventions such as US-guided injections [1]. Overall, MSK US imaging is indispensable for accurate, timely diagnosis, effective management of a wide range of musculoskeletal conditions, and the study of different anatomical structures [1].

This imaging modality offers high-resolution visualization of soft tissues, including muscles, tendons, ligaments, nerves, joints and fasciae [1], providing detailed anatomical and pathological information. Its real-time capability enables dynamic assessment of structures during movement, which is particularly useful for evaluating tendon gliding, detecting subluxations, diagnosing impingements, etc. [2,3].

In this era of high-definition US imaging, another structure that is garnering increased attention is the ankle retinacula [4,5]. Recent research underscores the critical role of the ankle retinacula in proprioceptive feedback and the functional stability of the ankle [6,7,8]. Retinacula are localized thickenings of the deep/muscular fascia that keep tendons in place during muscle contraction, thereby preventing bowstringing [4]. The high mechanical stress in the ankle during sport activities, coupled with frequent acute trauma and repetitive microtrauma, leads to common yet underreported disorders of the ankle retinacula in clinical practice [9,10,11]. Ultrasound (US) imaging is particularly effective in visualizing ankle retinacula due to their superficial location and the high spatial resolution of US imaging [9]. Moreover, dynamic US imaging can effectively demonstrate intermittent tendon dislocations caused by retinacular failure [9,10,11]. The retinacula are anatomically structured into three unique histological layers: an inner gliding layer rich in hyaluronic acid-secreting cells; a substantial middle layer composed of collagen bundles, fibroblasts and elastin fibers all interwoven; and an outer layer of loose connective tissue containing vascular channels [8]. Injuries to the retinacula are predominantly linked to ankle sprains, generally induced by a sudden and vigorous traction of the neighboring tendons caused by intense muscle contraction [10]. Although less frequent, microtraumatic lesions of the retinacula can also arise in the context of sport activities [10].

Despite the clear advantages of US imaging in diagnosing ankle retinacula injuries, there is a notable gap in the literature concerning a comprehensive overview of the methodology, use and reliability of US imaging in this context. Existing studies often highlight the immediate diagnostic capabilities of US, yet they seldom provide an in-depth analysis of the protocols, parameters and techniques that enhance its effectiveness and reliability. Addressing these gaps through detailed methodological reviews and reliability assessment could significantly advance the clinical utility of US imaging, offering physicians a more thorough understanding of its strengths and limitations in diagnosing and managing ankle retinacula injuries. Hence, this comprehensive review was performed aiming to firstly investigate the use and the methodology of US imaging to assess pathologic and healthy ankle retinacula, and secondly to assess the reliability of US imaging in ankle retinacula assessment.

## 2. Materials and Methods

### 2.1. Data Sources

We performed a systematic literature review adhering to the Preferred reporting Items for Systematic Reviews and Meta-Analyses (PRISMA) guidelines. This systematic review protocol is registered in Open Science Framework registries with the registration https://doi.org/10.17605/OSF.IO/UVBRC (accessed on 2 June 2024). The search of the literature was guided by PICO (Problem/Patient; Intervention/Indicator; Comparison and Outcome) (Table 1).

Our search was conducted on the PubMed and Web of Science databases, covering publications from their inception until May 2024. Additionally, we meticulously examined the references of the included studies to identify any further relevant publications. The MeSH keywords employed were “Ankle Retinacula”, “Foot Retinacula”, “Superior extensor retinaculum”, “Inferior extensor retinaculum”, “peroneal retinaculum”, “superior peroneal retinaculum”, “inferior peroneal retinaculum”, “flexor retinaculum”, “Ultrasound Imaging”, “Ultrasound”, “Ultrasonography” and “Ultrasound examination”. The search strategy was structured as follows: (“Ankle retinacula”) OR (“ankle retinacula” AND “Ultrasound Imaging”) OR (“ankle retinacula” AND “Ultrasound”) OR (“ankle retinacula” AND Ultrasonography”) OR (“ankle retinacula” AND “Ultrasound examination”) OR (“Foot Retinacula”) OR (“Foot Retinacula” AND “Ultrasound Imaging”) OR (“Foot Retinacula” AND “Ultrasound”) OR (“Foot Retinacula” AND Ultrasonography”) OR (“Foot Retinacula” AND “Ultrasound examination”) OR (“Superior extensor retinaculum”) OR (“Superior extensor retinaculum” AND “Ultrasound Imaging”) OR (“Superior extensor retinaculum” AND “Ultrasound”) OR (“Superior extensor retinaculum” AND Ultrasonography”) OR (“Superior extensor retinaculum” AND “Ultrasound examination”) OR (“Inferior extensor retinaculum”) OR (“Inferior extensor retinaculum” AND “Ultrasound Imaging”) OR (“Inferior extensor retinaculum” AND “Ultrasound”) OR (“Inferior extensor retinaculum” AND Ultrasonography”) OR (“Inferior extensor retinaculum” AND “Ultrasound examination”) OR (“peroneal retinaculum”) OR (“peroneal retinaculum” AND “Ultrasound Imaging”) OR (“peroneal retinaculum” AND “Ultrasound”) OR (“peroneal retinaculum” AND Ultrasonography”) OR (“peroneal retinaculum” AND “Ultrasound examination”) OR (“superior peroneal retinaculum”) OR (“superior peroneal retinaculum” AND “Ultrasound Imaging”) OR (“superior peroneal retinaculum” AND “Ultrasound”) OR (“superior peroneal retinaculum” AND Ultrasonography”) OR (“superior peroneal retinaculum” AND “Ultrasound examination”) OR (“inferior peroneal retinaculum”) OR (“inferior peroneal retinaculum” AND “Ultrasound Imaging”) OR (“inferior peroneal retinaculum” AND “Ultrasound”) OR (“inferior peroneal retinaculum” AND Ultrasonography”) OR (“inferior peroneal retinaculum” AND “Ultrasound examination”) OR (“flexor retinaculum”) OR (“flexor retinaculum” AND “Ultrasound Imaging”) OR (“flexor retinaculum” AND “Ultrasound”) OR (“flexor retinaculum” AND Ultrasonography”) OR (“flexor retinaculum” AND “Ultrasound examination”).

### 2.2. Study Selection and Searchers

Studied were included if they (1) used US imaging in the evaluation of ankle retinacula; (2) used US imaging to diagnose a pathology of ankle retinacula; (3) were published and conducted within the Table 1 criteria; and (4) were published in the English language. Exclusion criteria were applied to filter out peripheral content, maintaining a focus on primary research while including review papers and case reports. The exclusion criteria were (1) papers centered on treatment; (2) studies focused on US-guided injections; (3) studies on surgery; (4) papers that did not discuss US imaging for ankle/foot retinacula; and (5) non-English publications.

**Figure 1:**
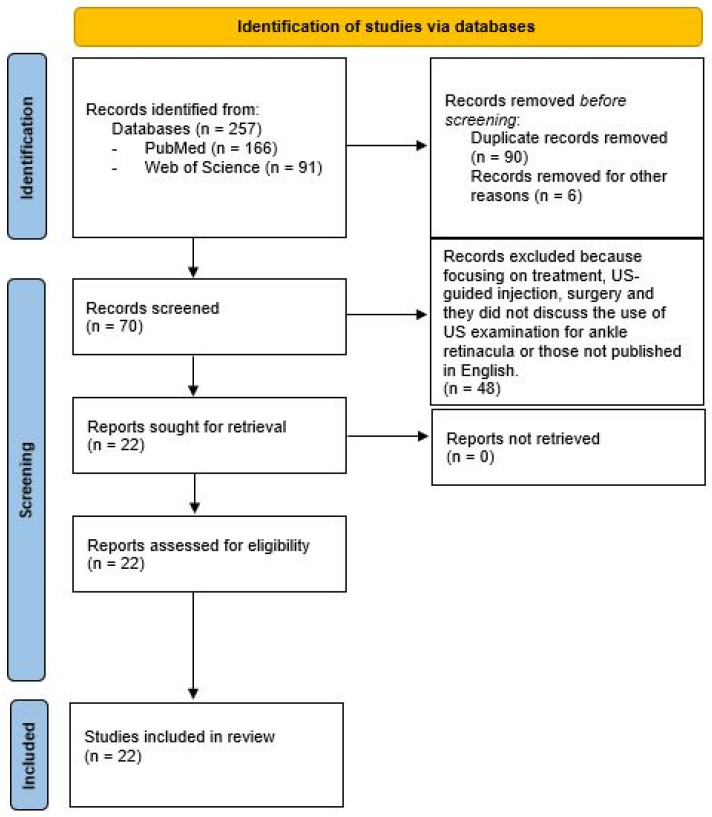
Flow chart of study selection.

Our screening process involved reviewing title and abstracts, followed by a full-text review of eligible studies. References were also checked to identify any additional publications. The literature search was conducted by one reviewer (N.P.) and verified by a senior researcher (C.P.) with ten years of experience. Any discrepancies were resolved through consensus among the authors (Figure 1).

### 2.3. Data Extraction

Data concerning these parameters were collected and analyzed:

- General characteristic of the paper: first author, year of publication, study design;
- Study population characteristics: number of patients or healthy volunteers, age, gender and ankle/foot retinacula status;
- Measurement methods: type of probe, type of US imaging, positions of patients or healthy volunteers;
- Reliability;
- Outcomes: evaluated parameters.

### 2.4. Risk of Bias

Two researchers assessed the quality of the studies, resolving any discrepancies through discussion. The evaluation of the papers utilized the Newcastle–Ottawa Scale (NOS), specifically tailored for both observational and case–control studies. Meanwhile, case-report studies were appraised using the JBI Critical Appraisal Checklist for Case Reports.

## 3. Results

A meticulous analysis was conducted on papers selected specifically for their relevance to the US imaging of ankle retinacula, encompassing both patient cases and studies involving healthy volunteers. The degree of consensus among the authors regarding the inclusion of the articles was remarkably high, demonstrated by a Cohen’s kappa value of 0.92. The essential attributes of the selected studies, spanning from 1974 to 2024, are succinctly outlined in Table 1 [10,11,12,13,14,15,16,17,18,19,20,21,22,23,24,25,26,27,28,29,30,31]. In total, 257 records underwent screening, resulting in the removal of 90 duplicates and the exclusion of six further records. Subsequently, the textual content of the remaining 70 potentially eligible papers was meticulously reviewed, leading to the exclusion of 48 papers that did not align with our predefined inclusion criteria. Ultimately, 22 studies met the criteria for inclusion. The schematic representation of our study process is depicted in Figure 1 and Table 2.

### 3.1. General Characteristics of Studies

According to their methodological design, most of the papers included in this review were review studies (n = 8) [5,13,14,21,22,24,25,26]. The other studies were cross-sectional studies (n = 2) [10,11], case report or case series (n = 6) [12,14,15,17,28,30], cohort studies (n = 2) [16,30], retrospective studies (n = 3) [18,23,24], cadaveric studies (n = 2) [19,20] and one pictorial essay (Table 3).

### 3.2. Typer of Population

Overall, the 22 papers in the current review included 269 participants, 67 healthy volunteers and 202 patients with pathological conditions at ankle retinacula; 155 male (58.82%) and 100 female (41.18%) individuals have been evaluated, with an average of 30.88 ± 15 years. The patients had rheumatoid arthritis, psoriatic arthritis, previous ankle sprains, ankle pain, posterior tendon dislocation, had been treated surgically for chronic peroneal tendon dislocation, inversion ankle trauma and painful snapping of peroneal tendons.

### 3.3. Assessed Ankle Retinacula

The papers included in this review addressed superior extensor ankle retinaculum [10,12,22,24,27,29], inferior extensor ankle retinaculum [12,18,22,27,29], superior peroneal retinaculum [5,11,13,14,15,18,21,23,25,26,27,29,30], inferior peroneal retinaculum [5,16,21,26,27,28] and flexor retinaculum [5,11,17,19,20,27].

### 3.4. US Equipment Features and Type of Probe

Across the studies included, multiple US devices were used, each equipped with linear array transducers [10,11,12,13,14,15,16,17,18,19,20,21,22,23,24,25,27,28,29,30] and one with a hockey stick probe [26]. The used frequencies varied from 9 to 20 MHz [14], 6 to 15 MHz [10,11,18], 12 to 18 MHz [11], 8 to 18 MHz [18,19,26], >9 MHz [22] and 3 to 12 MHz [24]. The US machines operated in different modes: B-mode [5,11,12,13,14,15,17,18,19,20,21,22,23,24,25,26,27,28,29,30], shear-wave elastography [16], sono-palpation [12] and power-Doppler [5,11,24]. Finally, some studies dynamically evaluated ankle retinacula [14,15,17,18,22,23,24,25,26,27,28,29].

### 3.5. Positioning of Patient and Type of Protocol

Different protocols were used to evaluated ankle retinacula: supine position of patient with flexed knee at 30 degrees [11]; supine position with extended knee [10,12,19,24]; standing on an articulated platelet with full weight bearing with ankle in neutral position, valgus 20° and varus 30° [16]; supine position with a slight dorsiflexion of the ankle [20]; supine position with the knee joint flexed and the ankle internally rotated [21]; and supine position, static: knee flexed and ankle internally rotated and dynamic: with a pillow under the calf in rest, with active and passive ankle dorsiflexion-eversion [22]. The position of the probe was in axial [29], longitudinal [29] and oblique [26] scans.

### 3.6. Parameters Evaluated with Measurements and Reliability

A multiplicity of parameters was evaluated such as integrity [14,15,21,26,28], stiffness [16], echogenicity [5,10,11,18,24,25,26,27,29,30], thickness [5,10,11,12,14,24,25,26,27] and anatomical locations [19,20,23]. Only 3 of 22 papers reported data about reliability [10,11,16].

### 3.7. Aims of Studies

A multiplicity of aims was reported. The main aim was the diagnosis [5,10,11,12,13,14,15,16,17,18,21,22,24,25,26,27,28,29,30] of different pathological conditions. Another aim was anatomical localization [19,20,23] through carrying out cadaveric studies.

### 3.8. Risk of Bias Assessment and Applicability Concern

The NOS score of the included studied articles is shown in Table 1 and Table 2. After evaluation by two researchers, the studies received an average NOS score of 5.1, indicative of intermediate quality studies (Table 4 and Table 5).

## 4. Discussion

This comprehensive review provided a detailed analysis of the layers of ankle retinacula, type of US equipment used, patient positioning protocols, evaluated parameters and reliability analyses. A total of 22 papers were identified that investigated ankle retinacula using advanced US imaging techniques. The studies varied in their methodological designs, including eight review studies [5,13,14,21,22,24,25,26], two cross-sectional studies [10,11], six case reports or case series (n = 6) [12,14,15,17,28,31], two cohort studies [16,29], three retrospective studies [18,23,24], two cadaveric studies [19,20] and one pictorial essay [27]. This variety reflects the comprehensive scope of the review. In total, the included studies comprised 269 participants, of which 67 were healthy volunteers and 202 were patients with pathological conditions affecting the ankle retinacula. The average age of the participants was 30.88 ± 15 years old. The review’s meticulous approach in combining diverse study designs and participant demographics provided a broad and nuanced perspective on the current state of research in ankle retinacula assessment through US imaging, highlighting both the advancements and the areas requiring further investigation.

**Figure 2:**
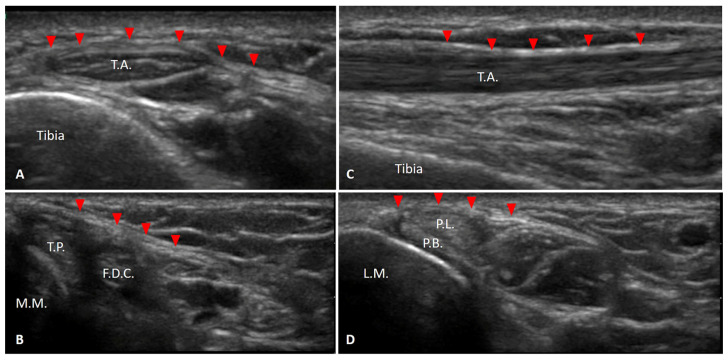
Ultrasound imaging of ankle retinacula. (**A**) Superior extensor retinaculum (short axis). (**B**) Flexor retinaculum. (**C**) Superior extensor retinaculum (long axis). (**D**) Superior peroneal retinaculum. T.A.: tibialis anterior tendon; T.P.: tibialis posterior tendon; F.D.C.: flexor digitorum comunis tendon; M.M.: medial malleolus; L.M.: lateral malleolus; Red triangles = retinaculum.

Ultrasound imaging offers an unparalleled level of detail when it comes to visualizing the retinacula of the ankle (Figure 2A–D).

This advanced imaging technique allows for the distinct identification and thorough assessment of each retinaculum, enabling clinicians to observe their normal anatomical features as well as any deviations that may indicate underlying pathologies. The retinacula of the ankle are characterized by a range of anatomical variants, which underscore the importance of numerous anatomical review studies aimed at precisely defining their anatomical localization. These studies [19,20,23] provide crucial insights that enhance our understanding of the variability, facilitating more accurate diagnosis and treatment of related conditions. The majority of data the focused on superior peroneal retinaculum [5,11,13,14,15,18,21,23,25,26,27,29,30], followed by superior extensor ankle retinaculum [5,10,12,22,24,27], superior peroneal retinaculum [5,11,13,14,15,18,21,23,25,26,27,29,30], inferior peroneal retinaculum [5,16,21,26,27,28], flexor retinaculum [5,11,17,19,20,27] and inferior extensor ankle retinaculum [5,12,18,22,27]. However, it is important to note that this level of detailed visualizations is particularly challenging for the inferior extensor retinaculum. The inferior extensor ankle retinaculum, due to its complex structure and location, presents more difficulties in US imaging compared to other retinacula. This complexity further justifies the need for extensive anatomical studies to ensure accurate identification and assessment using US imaging.

The authors of the included studies used a multiplicity of US devices and different types of transducers. Moreover, they were evaluated mainly with linear array transducers [10,11,12,13,14,15,16,17,18,19,20,21,22,23,24,25,27,28,29,30,31] and to a small extent with a hockey stick probe [26]. Regarding the frequency and depth of acquisition, the majority of papers examined reported frequency ranges for the US transducers, typically averaging from 6 to 18 MHz. B-mode was the most commonly used across the majority of the studies, followed by power-Doppler [5,11,24], in static and dynamic modalities [14,15,17,18,22,23,24,25,26,27,28,29], shear-wave elastography [16] and sometimes sono-palpation [12]. The comprehensive utilization of various US devices and transducers, as well as the frequency ranges employed in these studies, highlights the versatility and adaptability of US examination in the assessment of ankle retinacula. The predominant use of linear array transducers is appropriate given their high resolution and suitability for superficial structures. However, the occasional use of hockey stick probes indicated an awareness of the need for more specialized US imaging in certain scenarios. The frequency range of 6 to 18 MHz aligns well with the requirements for US imaging of the detailed structures of the ankle retinacula, providing a balance between penetration depth and resolution. The use of B-mode as the primary imaging modality is expected, given its efficacy in structural assessment, while the supplementary use of power-Doppler, shear-wave elastography and sono-palpation demonstrates a thorough approach to capturing both anatomical and functional information.

The ultrasound ankle retinacula examinations were predominantly in the supine position with an extended knee [10,12,19,24], although the supine position with the knee flexed to varying degrees was widely used [10,12]. Some authors reported standing on an articulated platform with full weight bearing and the ankle in a neutral position allowing for 20° of valgus and 30° of varus [16]. The variety of patient positionings during the US examinations, including both supine and wight-bearing positions, reflects the need to understand the retinacula under different physiological conditions. While the supine positions, both in extended and flexed knee, provide essential baseline information, the standing position on an articulated platform with full weight bearing introduced a dynamic aspect that may reveal additional insights into the functional status of the ankle retinacula under load. Overall, the methodology described in these studies reflects a robust and well-rounded approach to US imaging of ankle retinacula, although the variation in devices, techniques and patient positions underscores the need for standardization in future research to ensure the consistency and comparability of the results. Moreover, the protocols ranged from passive positioning to active tasks to assess the integrity of ankle retinacula [14,15,21,26,28]. This approach ensures a comprehensive assessment that can better inform clinical practice by capturing the retinacula’s behavior under various conditions, thereby offering more complete understanding of their functional capabilities and potential pathological changes.

The US parameters assessed included thickness [5,10,11,12,14,24,25,26,27], stiffness [16] and echogenicity [5,10,11,18,24,25,26,27,29,30]. For the superior extensor retinaculum, Pirri et al. reported an average of 0.9 ± 0.45 mm in healthy volunteers and 1.3 ± 0.5 mm for the side of previous ankle sprains in football players [10]. For superior peroneal retinaculum, Forien et al. showed a thickness of 0.6 ± 0.12 mm in rheumatoid arthritis patients and 0.71 ± 0.27 mm for psoriatic arthritis patients [11]; for flexor retinaculum, they reported a thickness of 0.64 ± 0.15 mm in rheumatoid arthritis patients and 0.96 ± 0.4 mm in psoriatic arthritis patients [11]. The detailed assessment of various US parameters such as thickness, stiffness and echogenicity, as demonstrated in the studies, underscores the capability of US imaging to provide critical insights into the structural and pathological conditions of ankle retinacula. The significant differences in retinacula thickness between healthy individuals and those with conditions like previous ankle sprains or arthritis emphasize the potential of US imaging as a diagnostic tool. However, these variations also highlight the need for a standardized measurement protocol to ensure consistency and reliability across different studies. The findings presented in this review pave the way for future research to refine and validate US parameters, ultimately enhancing their application in clinical practice.

Only three studies assessed the reliability of ankle retinacula US measurements, with intra-rater reliability consistently reported as good to excellent agreement. Pirri et al. [10] reported an excellent intra-rater reliability in the superior extensor retinaculum with an average of ICC: 0.91 (0.88–0.94). In the vertical positioning of a patient with the valgus and varus position for inferior extensor retinaculum, Rougereau et al. [16] reported a normal ICC: 0.90 (0.81–0.94); valgus 20° ICC: 0.86 (0.76–0.92); varus 30° ICC: 0.89 (0.79–0.94). The studies reviewed provide compelling evidence supporting the reliability of US measurements for ankle retinacula, particularly highlighting the strong intra-rater reliability. The results reported by Pirri et al. [10] and Rougereau et al. [16] demonstrate consistent and high ICC values across different conditions, underscoring the robustness of these measurements in clinical practice. This consistency is crucial for the diagnostic and therapeutic use of US in evaluating the ankle retinacula, suggesting that these techniques can be reliably employed across various patient positions and conditions. However, the limited number of studies underscores the need for further research to confirm these findings and expand the evidence base, particularly in diverse patient populations and settings.

Ultrasound imaging excels in identifying and characterizing a variety of pathological conditions affecting the retinacula:

- Traumatic injuries: Acute trauma can lead to partial or complete tears of the ankle retinacula, which appear as hypoechoic disruptions within normally hyperechoic retinacular layers [5,13,14,21,22,24,25,26]. US imaging can also reveal associated tendon dislocation or subluxation. For example, Hosack et al. reported that peroneal tendon dislocation or subluxation typically results from injury to the superior peroneal retinaculum, affecting the tendons in the retromalleolar groove; this is classified using the modified Eckert and Davies system.
- Chronic conditions: Overuse or repetitive strain can cause chronic thickening of retinacula, often accompanied by calcifications. US imaging effectively identifies these changes, aiding in the diagnosis of conditions such as stenosing tenosynovitis [5,10,12,13,14,21,22,24,25,26].
- Inflammatory and degenerative changes: Inflammatory conditions may present as increased vascularity on Doppler imaging, while degenerative changes can lead to inhomogeneous echotexture and loss of the normal fibrillar pattern [5,11,13,14,21,22,24,25,26]. Forien et al. showed that US abnormalities of ankle flexor retinacula were more frequent and specific in psoriatic arthritis patients than in rheumatoid arthritis patients, suggesting that US examination of ankle flexor retinacula can help distinguish between the two conditions [11].

One the most significant advantages of ankle retinacula US examination over other imaging modalities is its dynamic assessment capability [3]. Real-time imaging allows clinicians to evaluate the ankle retinacula and associated tendons during movement, providing critical insights into functional abnormalities.

- Tendon instability: dynamic US imaging can demonstrate subluxation or dislocation of tendons, particularly in the context of peroneal retinacula pathology [3,5,11,13,14,15,18,21,23,25,26,27,29,30,31].
- Assessment of tendon-gliding mechanism: evaluating tendon movement relative to the ankle retinacula during active and passive maneuvers helps in diagnosing and understanding the functional impact of various retinacula pathologies [13,14,21,22,24,25,26,29].

It is crucial to recognize several limitations in the referenced studies. One of the primary issues is that the majority of these studies were reviews rather than original research, which inherently limits the strength of the conclusions that can be drawn. Another significant concern is the heterogeneity of the data across these studies, which precludes their synthesis and makes it difficult to draw consistent, overarching conclusions. Given these limitations, it is evident that future research should prioritize conducting more clinical trials. These trials are essential to validate the proposed parameters and ensure their reliability and applicability in daily clinical practice. Only through such rigorous, original research can we hope to establish a solid foundation of evidence that can be effectively utilized in practical settings.

## 5. Conclusions

Ultrasound imaging is invaluable in assessing the ankle retinacula of ankle and foot, offering detailed visualization of both normal and pathological conditions. Understanding the normal sonographic appearance of these structures and recognizing common pathologic changes are essential for accurate diagnosis and appropriate management of ankle retinacula disorders. Dynamic US further enhances the assessment by allowing real-time evaluation of ankle retinacula function, particularly in cases of suspected instability or dislocation. In the end, future research should focus on clinical trials to validate parameters and ensure their reliability in clinical practice.

## Figures and Tables

**Table 1:** Description of the PICO (P = Population, I = Intervention, C = Comparison, O = Outcome) elements.

Population	Patients or healthy volunteers who underwent ultrasound imaging of ankle retinacula
Intervention	Ultrasound imaging
Comparison	Ultrasound imaging of different types of ankle retinacula
Outcome	Parameters of thickness, echogenicity, stiffness, displacement

**Table 2:** Papers on ultrasound imaging of ankle/foot retinacula.

Authors and Year	Type of Paper	Number of Participants	Type of Patients	Sex	Age	Type of Anatomical Structure	Type of Probe (Frequency)	Type of US Imaging	Position of the Patient	Position of the Probe	Parameters Evaluated with the Measurements	**Thickness Measurement**	**Reliability**	**Aim**
Pirri C (2024) [10]	Cross-setional study	50	- 25 healthy subjects - 25 football players with previous ankle sprains	50 M	29 ± 11 y.	Superior extensor retinaculum	6–15 MHz linear transducer	B-mode	Supine position	Ultrasound transducer was positioned parallel to the tibia, approximately 0.5 cm lateral to the medial tibial crest, above the tibia–talar joint, and lateral to the anterior border of the distal tibia up to the lateral malleolus	Thickness and echogenicity	SEAR thickness showed that in group 1, along the longitudinal and transversal axes, it was thicker on the previous multiple ankle sprains side (long. = 1.3 ± 0.44 mm; transv. = 1.33 ± 0.51 mm) than on the healthy side (long. = 0.9 ± 0.4 mm; transv. = 0.92 ± 0.44 mm)	0.92 (0.88–0.96)	To determine an ultrasonographic parameter or difference that can quantify the superior extensor ankle retinaculum status in football players with previous multiple ankle sprains compared with healthy volunteers.
Forien M (2024) [11]	Cross-sectional study	80	- RA (rheumatoid arthritis); - PsA (psoriatic arthritis)	33 M 47 F	>18 y.	Superior peroneal retinaculum and the flexor retinaculum	12–18 MHz linear transducer	B-mode and power Doppler	Supine position with the knee flexed at 30 degrees to assess the ankle	-	Thickness, echogenicity and vascularization of SPR and FR	- SPR: 0.60 ± 0.12 mm (RA); 0.71 ± 0.27 mm (PsA). - FR: 0.64 ± 0.15 mm (RA); 0.96 ± 0.40 mm (PsA).	r = 0.63, 95% CI [0.41–0.78]; [95% CI 0.0–0.0]	To compare the ultrasonography (US) assessment of the retinacula of ankles in patients with rheumatoid arthritis (RA) and psoriatic arthritis (PsA).
Pirri C. (2023) [12]	Case report	1	right ankle pain	M		Superior extensor retinaculum and inferior extensor retinaculum		B-mode and sonopalpation	-	-	Thickness	2.05 mm	-	-
Hosack T. (2023) [13]	Review	-	-	-	-	Superior peroneal retinaculum	-	-	-	-	-	-	-	-
Fritz B (2023) [14]	Review	1	lateral ankle pain and snapping sensation	M	54 y.	Superior peroneal retinaculum	High frequency transducers of 9 MHz to 20 MHz.	B-mode dynamic	-	Strict perpendicular to the tendons on long- and short-axis	Integrity and thickness	-	-	To report the MR Imaging–Ultrasonography Correlation of Acute and Chronic Foot and Ankle Conditions.
Grandberg C. (2022) [15]	Case report	1	pain and locking of the lateral side of the left foot 2 years before; no trauma.	F	25 y.	Superior peroneal retinaculum	Linear probe	B-mode dynamic	-	-	Integrity	-	-	To report a case of subluxation of the peroneus brevis tendon, with no apparent traumatic cause, in which there was a need for a surgical approach after the failure of conservative treatment.
Rougereau G. (2022) [16]		20	healthy	10 M 10 F	Mean aged 22.2 (range from 22 to 31 years)	Inferior extensor retinaculum (IER)	8 MHz linear probe	SWE	Standing on an articulated platelet with full weight bearing with ankle in neutral position, valgus 20°, and varus 30°	Vertically anterior and inferior to the lateral malleolus, opposite the tarsal sinus	Stiffness	-	- Normal: 0.90 [0.81–0.94]; - Valgus 20°: 0.86 [0.76–0.92]; - Varus 30°: 0.89 [0.79–0.94].	To evaluate the stiffness of the inferior extensor retinaculum (IER) using shear-wave elastography (SWE) in neutral and varus positions in healthy adults and to assess the reliability and reproducibility of these measurements.
Zannoni S. (2022) [17]	Case report	1	posterior tibial tendon dislocation.	-	-	Flexor retinaculum	-	B-mode and Dynamic	-	-	-	-	-	To report a case of traumatic subluxation of the posterior tibial tendon, illustrating imaging findings and surgical technique.
Drakonaki EE (2021) [18]	Retrospective study	63	with available ankle US, MR and CT images	38 F 25 M	mean age 32.7, range 18–58 years	Superior peroneal retinaculum and inferior extensor retinaculum	6–15 MHz and 8–18 MHz probes	B-mode and dynamic	-	-	Echogenicity	-	-	This imaging anatomy study was aimed at detecting anatomical variations and potential interconnections of the superior peroneal retinaculum to other lateral stabilizing structures.
Iborra Á (2019) [19]	Prospective study	12	cadaveric specimens	-	-	Flexor retinaculum	8–17 MHz linear transducer	B-mode	Supine position on the operating table	Longitudinal and transverse planes were used to delineate the anatomic boundaries of the proximal and distal tarsal tunnels.	Anatomical localization	-	-	To determine whether ultrasound (US)-guided surgery is a viable type of surgery for performing an effective release/decompression of the constricting structures that are responsible for focal nerve compression in tarsal tunnel syndrome.
Fernández-Gibello A (2019) [20]		10	cadaveric fresh/frozen feet	4 M 6 F	-	Flexor retinaculum	13 MHz linear transducer	B-mode	Supine position, with a slight dorsiflexion of the ankle	Long axis at the DM-line	Anatomical localization	-	-	To provide a safe ultrasound-guided minimally invasive surgical approach for a proximal tarsal tunnel release concerning nerve entrapments.
Draghi F (2018) [21]	Review	-	-	-	-	Superior and inferior peroneal retinacula	High-frequency linear array transducer	B-mode	Supine on the examination table, the knee joint flexed and the ankle internally rotated	Long axes by placing the transducer in an oblique plane according to their course	Integrity	-	-	To provide an overview of the anatomic basis for peroneal intrasheath instability and provide physicians with guidelines for its ultrasound assessment.
Kumar Y (2017) [22]	Review	-	-	-	-	Superior peroneal retinaculum (SPR) and inferior peroneal retinaculum (IPR)	A high-resolution ultrasound probe (>9 MHz)	B-mode and dynamic	Supine position - static: knee flexed and ankle internally rotated; - dynamic: with a pillow under the calf in rest, active and passive ankle dorsiflexion-eversion	SPR: transducer oriented in the axial oblique plane	-	-	-	To discuss the role of dynamic ultrasound and kinematic MRI for the evaluation of peroneal tendons will.
Choufani C (2017) [23]	Retrospective study (Level IV)	17	treated surgically for chronic fibular ten-don dislocation at a single center	9 M 8 F	32.6 ± 9.7 years (range, 18–52 years)	Superior fibular retinaculum	Linear probe	Dynamic US	-	-	Anatomical localization	-	-	To evaluate the outcomes of this surgical technique as assessed by a functional score and dynamic ultrasonography.
Ding J (2016) [24]	Retrospective review	7	child after inversion trauma of the ankle	4 M 3 F	mean age 13.4 years; age range 10–15 years	Superior extensor retinaculum (SER)	3–12 MHz transducer	B-mode, dynamic US (if no acute case) and Power -Doppler	Supine position	Coronal and anterior sagittal scans of the distal fibula and anterior sagittal scans focused on the growth plate	Thickness and echogenicity	-	-	To describe a new sonographic feature for a traumatic lesion of the ankle in children.
Pesquer L (2016) [25]	Review	-	-	-	-	Superior peroneal retinaculum	Linear probe	B-mode, Dynamic US	-	-	Thickness and echogenicity	-	-	To describe the anatomic and physiologic bases for peroneal instability and to heighten the role of dynamic ultrasound in the diagnosis of snapping.
Taljanovic MS (2015) [26]	Review	-	-	-	-	Superior peroneal retinaculum (SPR) and inferior peroneal retinaculum (IPR)	8- to 18 MHz linear “hockey stick” transducer	B-mode and Dynamic US	-	In the axial oblique plane	Thickness, echogenicity and integrity	-	-	To review the normal anatomy of the peroneal tendons at US and MR imaging, discuss different types of peroneal tendon injuries and ankle instability seen at MR imaging and US, and review treatment options.
Precerutti M (2013) [27]	Pictoral essay	-	-	-	-	Retinacula of the ankle	Linear probe	B-mode	-	-	Thickness and echogenicity	-	-	An intimate knowledge of the ankle sonography.
Staresinic M (2013) [28]	Case series	3	professional soccer players	M	20, 23 and 28 years old	Inferior peroneal retinaculum (IPR)	Linear probe	B-mode	-	-	Integrity	-	-	To present an assessment, diagnostic algorithm and new therapeutic option for the distal dislocation of the long peroneal tendon due to isolated inferior peroneal retinaculum (IPR) tear.
Demondion X (2010) [5]	Review	-	-	-	-	Superior and inferior extensor retinacula; superior and inferior peroneal retinaculum; flexor retinaculum.	Linear probe	B-mode and vascular Doppler	-	-	Thickness and echogenicity	-	-	To describe the anatomy and the injuries of the retinacula of the ankle and foot.
Raikin SM (2008) [29]	Cohort study	56	painful snapping of the peroneal tendons posterior to the fibula	-	-	Superior peroneal retinaculum	A high-frequency linear array transducer	B-mode and dynamic US	-	Axial and longitudinal ultrasound scan of the ankle	-	-	-	To identify a new subgroup of patients with intra-sheath subluxation of these tendons within the peroneal groove and with an otherwise intact retinaculum.
Karlsson J (2002) [30]	Case report	1	2 weeks of progressivepain and swelling of his left ankle.	M	56 y.	Superior peroneal retinaculum	10 MHz compact linear probe	B-mode	-	-	Echogenicity and position	-	-	To discuss the ultrasonographic appearance of peroneus longus and peroneus brevis tendon splits and the mechanism of injury.

y. = years; F = female; M = male; Ns = non specified.

**Table 3:** General characteristics of 22 papers included in our analysis.

Type of Study	N
Review	8
Cross-sectional studies	2
Case report or case series	6
Cohort study	2
Retrospective study	3
Cadaveric study	2
Pictorial essay	1

**Table 4:** Study of quality assessment using Newcastle–Ottawa scale for observational studies.

References	Selection				Comparability (Matched Analysis)	Assessment of Outcome	Outcomes	Adequacy of Follow-Up of Cohorts	NOS Score
	Consecutive or Obviously Representative Series of Cases	Representativeness of Exposed Cohort	Ascertainment of Exposure	Demonstration That Outcome of Interest Was Not Present at the Start of Study			Follow Up Long Enough for the Outcome		
Pirri [10]	**	*	*	-	**	**	-	-	8
Forien [11]	**	*	*	-	**	*	-	-	8
Rougereau [16]	*	*	*	*	*	*	-	-	6
Drakonaki [18]	*	*	*	*	*	*	-	-	6
Iborra [19]	*	*	-	*	-	*	-	-	4
Fernandez-Gibello [20]	-	-	*	*	-	*	-	-	3
Choufani [23]	-	-	*	-	-	*	-	-	2
Ding [26]	-	*	-	-	-	*	-	-	2
Raikin [29]	*	*	*	*	*	*	-	-	6

*,** Each asterisk represents if individual criterion within the subsection was fullfilled.

**Table 5:** Study of quality assessment using JBI Critical Appraisal Checklist for Case Reports.

References	Were Patient’s Demographic Characteristics Clearly Described?	Was the Patient’s History Clearly Described and Presented as a Timeline?	Was the Current Clinical Condition of the Patient on Presentation Clearly Described?	Were Diagnostic Tests or Assessment Methods and the Results Clearly Described?	Was the Intervention(s) or Treatment Procedure(s) Clearly Described?	Was the Post-Intervention Clinical Condition Clearly Described?	Were Adverse Events (Harms) or Unanticipated Events Identified and Described?	Does the Case Report Provide Takeaway Lessons?
Pirri [12]	Y	Y	Y	Y	Y	Y	-	Y
Grandberg [15]	Y	Y	Y	Y	-	-	-	Y
Zannoni [17]	Y	Y	Y	Y	-	-	-	Y
Staresini [28]	Y	Y	Y	Y	-	-	-	Y
Karlson [30]	Y	Y	Y	Y	-	-	-	Y

## Data Availability

The data presented in this study are available upon request from the corresponding authors. The data are not publicly available due to privacy.
